# Comparison of diagnostic accuracy in sepsis between presepsin, procalcitonin, and C-reactive protein: a systematic review and meta-analysis

**DOI:** 10.1186/s13613-017-0316-z

**Published:** 2017-09-06

**Authors:** Chin-Chieh Wu, Hao-Min Lan, Shih-Tsung Han, Chung-Hsien Chaou, Chun-Fu Yeh, Su-Hsun Liu, Chih-Huang Li, Gerald N. Blaney, Zhen-Ying Liu, Kuan-Fu Chen

**Affiliations:** 1 Department of Emergency Medicine, Chang Gung Memorial Hospital, Keelung, Taiwan; 2 School of Medicine, Chang Gung University, Taoyuan, Taiwan; 3 Department of Emergency Medicine, Chang Gung Memorial Hospital, Linkou, Taiwan; 4 Division of Infectious Diseases, Department of Internal Medicine, Chang Gung Memorial Hospital at Linkou, Chang Gung University College of Medicine, Taoyuan, Taiwan; 5 Department of Family Medicine, Chang Gung Memorial Hospital, Linkou, Taiwan; 6 Clinical Informatics and Medical Statistics Research Center, Chang Gung University, 5 Fu-Shin Street, Gueishan Village, Taoyuan, 333 Taiwan; 7 Community Medicine Research Center, Chang Gung Memorial Hospital, Keelung, Taiwan

**Keywords:** Presepsin, sCD14, Sensitivity, Specificity, Meta-analysis, Sepsis, Adults

## Abstract

**Background:**

The soluble cluster of differentiation 14 (or presepsin) is a free fragment of glycoprotein expressed on monocytes and macrophages. Although many studies have been conducted recently, the diagnostic performance of presepsin for sepsis remains debated. We performed a systematic review and meta-analysis of the available literature to assess the accuracy of presepsin for the diagnosis of sepsis in adult patients and compared the performance between presepsin, C-reactive protein (CRP), and procalcitonin (PCT).

**Methods:**

A comprehensive systemic search was conducted in PubMed, EMBASE, and Google Scholar for studies that evaluated the diagnostic accuracy of presepsin for sepsis until January 2017. The hierarchical summary receiver operating characteristic method was used to pool individual sensitivity, specificity, diagnostic odds ratio (DOR), positive likelihood ratio (PLR), negative likelihood ratio (NLR), and area under the receiver operating characteristic curve (AUC).

**Results:**

Eighteen studies, comprising 3470 patients, met our inclusion criteria. The pooled diagnosis sensitivity and specificity of presepsin for sepsis were 0.84 (95% CI 0.80–0.87) and 0.76 (95% CI 0.67–0.82), respectively. Furthermore, the pooled DOR, PLR, NLR, and AUC were 16 (95% CI 10–25), 3.4 (95% CI 2.5–4.6), 0.22 (95% CI 0.17–0.27), and 0.88 (95% CI 0.85–0.90), respectively. Significant heterogeneity was found in both sensitivities (Cochrane *Q* = 137.43, *p* < 0.001, *I* ^2^ = 87.63%) and specificities (Cochrane *Q* = 180.76, *p* < 0.001, *I* ^2^ = 90.60%). Additionally, we found no significant difference between presepsin and PCT (AUC 0.87 vs. 0.86) or CRP (AUC 0.85 vs. 0.85). However, for studies conducted in ICU, the pooled sensitivity of presepsin was found to be higher than PCT (0.88, 95% CI 0.82–0.92 vs. 0.75, 95% CI 0.68–0.81), while the pooled specificity of presepsin was lower than PCT (0.58, 95% CI 0.42–0.73 vs. 0.75, 95% CI 0.65–0.83).

**Conclusion:**

Based on the results of our meta-analysis, presepsin is a promising marker for diagnosis of sepsis as PCT or CRP, but its results should be interpreted more carefully and cautiously since too few studies were included and those studies had high heterogeneity between them. In addition, continuing re-evaluation during the course of sepsis is advisable.

## Background

Sepsis, a life-threatening disease, contributes to more than twenty thousand deaths in the USA each year, accounting for almost 1–2% of all patients admitted to the hospitals, and as much as 25% of intensive care unit (ICU) admissions [1]. Typically, patients with sepsis can be treated efficiently with early intravascular fluid and antibiotics in the ICU to avoid mortality development. However, it is difficult to decide early on whether to apply these methods due to the existence of non-infectious systemic inflammatory response syndrome (SIRS) [2]. Therefore, identifying a biomarker that can efficiently distinguish sepsis from non-infectious SIRS has become an important topic.

Recently, some studies investigated candidate biomarkers to detect sepsis reported such as procalcitonin (PCT), C-reactive protein (CRP), lipopolysaccharide-binding protein (LBP), interleukins, provasopressin, and myeloid cells expressing triggering receptor-1 (TREM-1) [3–7]. However, none of them have been proven to be accurate enough to distinguish sepsis from SIRS. The Surviving Sepsis Campaign further ‘weakly’ recommended the measurement of procalcitonin levels to be used to support shortening the duration or discontinuation of antimicrobial therapy in sepsis patients under low quality of evidence [8]. Although CRP and PCT are the preferred biomarkers to be used in clinical context currently [9, 10], some issues for their diagnostic accuracy still remain unsolved, which prevent clinicians from starting or withholding antimicrobial therapy. A previous review revealed that CRP performs relatively inaccurately in the diagnostic tasks of sepsis compared with PCT [11]. The results from three published reviews indicated that the sensitivity and specificity of both CRP (ranged from 35 to 100% and from 18 to 84%, respectively) and PCT vary (ranged from 42 to 100% and from 48 to 100%, respectively) [10–12]. In addition, studies suggested that the CRP level increases in 4–6 h and reaches the peak in 48–72 h after the inflammatory onset [13], while PCT level increases in 8–24 h and reaches the peak later than 24 h [14]. Therefore, both PCT and CRP might be still not reliable enough as early indicators for sepsis used in the clinical context.

CD14 is a free fragment of glycoprotein expressed on monocytes and macrophages. It is a receptor of lipopolysaccharide–lipopolysaccharide-binding protein (LPS–LBP) complexes, transducing the endotoxin signal from bacterial infection through the Toll-like receptor-4 with the help of thinositol lipid structure [15]. Its soluble form, soluble CD14 (sCD14), is produced from cell secretion or when membrane-bound, CD14 (mCD14) detaches from cells such as phagocytes. The N-terminal fragments of 13 kDa consist of sCD14 subtype (sCD14-ST), also called presepsin, are closely related to mediating the immune response to LPS [16] and could be detected easier than mCD14 in the blood. Similar to other reported biomarkers, the distribution of presepsin values is slightly different, with a small overlap between healthy controls (294.2 ± 121.4 pg/ml) and septic patients (817.9 ± 572.7 pg/ml) [17]. Moreover, the level of presepsin typically increases within 2 h and reaches the peak in 3 h after infection [14]. By using the chemiluminescence enzyme immunoassay as detecting tool, the result can be available in 1.5 h [18]. The above evidence indicates that presepsin might be a better biomarker for sepsis during the early stage of sepsis than in later stages.

Recently, more clinical trials assessing the diagnostic accuracy of presepsin have been published. Most reported better results compared to other biomarkers. Therefore, we conducted this systematic review and meta-analysis not only to pool the results from relevant studies, but also to compare the diagnostic accuracy of presepsin with other biomarkers in diagnosing sepsis. We aimed to generate a more comprehensive understanding of the diagnostic performance and potential influence factors of presepsin in distinguishing sepsis from non-infectious SIRS.

## Methods

This study was performed and reported in accordance with the relevant reporting guideline (Preferred Reporting Items for Systematic Reviews and Meta-Analyses, PRISMA) [19].

### Search strategy and literature selection

We developed a protocol prior to conducting this systematic review and meta-analysis. A comprehensive systemic search was carried out in PubMed and EMBASE for studies evaluating the diagnostic accuracy of presepsin for sepsis until January 2017. The following search terms were used: [(‘Systemic Inflammatory Response Syndrome’ OR ‘SIRS’) AND ‘Sepsis’] AND (‘Early Diagnosis’ OR ‘Diagnosis’) AND (‘sCD14’ OR ‘presepsin’) AND ‘adult’ in PubMed and [sensitivity OR diagnostic AND accuracy:1nk OR diagnostic AND (‘sepsis’/exp OR sepsis) AND (‘sCD14’ OR sCD14) OR (‘presepsin’ OR presepsin) AND (English)/lim] in EMBASE, to search for original, English language, research articles that studied the diagnostic accuracy between septic and non-septic in adult patients. Additionally, we also conducted searches on Google Scholar and checked the reference lists to avoid potential missingness.

Two reviewers (CCW and HML) independently screened and decided the inclusion of the studies in the review after removing duplicated references. The inclusion criteria were: (1) sepsis-related studies; (2) diagnostic instead of prognostic studies: i.e. diagnosing sepsis instead of predicting mortality; and (3) articles in English. On the other hand, studies were excluded according to the following criteria: (1) non-sepsis-related studies; (2) non-diagnostic studies; (3) non-original studies: e.g. literature review, editorial piece; (4) studies with no performance parameters given (i.e. sensitivity, specificity and 2 × 2 contingency tables); and (5) non-blood specimen. Agreement regarding study inclusion between the reviewers was assessed using the Cohen’s kappa statistic [20].

### Data extraction

All relevant information, such as study setting, material and method, statistical method, and the results, was extracted independently via a piloted electrical form (Microsoft Access) by two authors (CCW and HML). The results of their extraction were double-checked to ensure the accuracy and all discrepant results were solved by consensus meetings. We transformed the numbers of true positive, false positive, false negative, and true negative based on the provided indices of sensitivity, specificity, and sample size values for statistical calculation. If any information was not provided, we contacted the authors by emails.

### Quality assessment

Two authors (CCW and HML) independently assessed the risk of bias of each study by the Quality Assessment of Diagnostic Accuracy Studies (QUADAS-2) checklist [21] recommended by the Cochrane collaboration for the quality assessment of diagnostic studies. The QUADAS-2 tool is constituted of four domains, including patient selection, index test, reference standard, and flow and timing. It assists authors of systematic reviews in rating the risk of bias and applicability in diagnostic accuracy from their studies. To judge the risk of bias of each study, signalling questions were provided. Agreement between the two reviewers for the assessment of methodological quality was evaluated using the Cohen’s kappa statistic [20]. All disagreements were solved by consensus meetings.

### Meta-analysis

All statistical tests were two-sided, and statistical significance was defined as *p* value <0.05. The Midas module for Stata 13.1 (Stata Corporation, College Station, TX, USA) was used for all statistical and meta-analysis. We used mada package in R (version 3.1.3) to do the bivariate binomial mixed-effect meta-regression model. Midas and the QUADAS modules for Stata were used for all graphical display of the quality of the included studies. Publication bias was tested by the Deek’s effective sample size funnel plots versus the diagnostic odds ratio.

The pooled sensitivity, specificity, diagnostic odds ratio (DOR), positive likelihood ratio (PLR), negative likelihood ratio (NLR), and the area under the receiver operating characteristic curve (AUC) were calculated based on the hierarchical summary receiver operating characteristic (HSROC) method [22] for meta-analysis of diagnostic test data. The advantage of this approach was that it could maximize the use of available data from each study, irrespective of the threshold used. Additionally, the respective summary receiver operating characteristic curves (SROC) [23] and AUC, irrespective of the different cut-off points used, were also conducted.

We checked heterogeneity of these included studies, as well as in different subgroups, to further evaluate the performance of presepsin. Heterogeneity can be caused by two types of effects, threshold and non-threshold. For threshold effects, the heterogeneity was calculated by the Spearman correlation coefficient (*ρ*) between logarithms of sensitivity and (1-specificity) and visual inspection from the SROC curve. Chi-square (*χ* ^2^) test, Cochrane *Q* test, and the *I* ^2^ metric were used for non-threshold effect heterogeneity [24]. A value of *I* ^2^ greater than 50% was considered significant heterogeneity. When heterogeneity was present, univariable meta-regression analyses using bivariate binomial mixed-effect model and subgroup analyses were performed to find the source of variability by potentially influencing factors in sensitivity and specificity, including country of study, patient sources, types of specimen, sample sizes, proportions of patients with sepsis, cut-off values, and different compositions of control groups. Additionally, some of our included studies were direct comparisons between presepsin, PCT, and CRP. Subgroup analyses were conducted to compare the diagnostic accuracy and performance between these three biomarkers. We also used the information on cut-off from each study to determine an optimal cut-off by maximizing the Youden index (sensitivity + specificity-1) in this diagnostic test accuracy reviews.

## Results

**Fig. 1:**
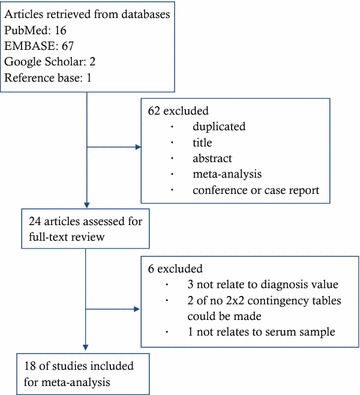
Flow chart of study identification, inclusion, and exclusion for meta-analysis

Our search in electronic databases yielded 86 published studies, 62 of which were excluded for various reasons based on screening the titles and abstracts (Fig. 1), leaving 24 studies that were assessed for full-text review. Among the 24 studies, we excluded another six studies; three were not related to diagnostic test, two did not report enough information to conduct a 2 × 2 contingency table, and one was not related to blood sample. Finally, 18 studies fulfilled our eligibility criteria and were included in the final meta-analyses [17, 25–41] (Fig. 1).

### Characteristics of included studies

The main characteristics and data of each included study are summarized in Tables 1 and 2. The 18 included studies were published between 2011 and 2017. Of the 3470 patients included in the 18 studies, 1904 (54.88%) were admitted to the emergency departments (ED), 783 (22.56%) were admitted to the ICU, and 783 (22.56%) were admitted in the ED and ICU. Among the 1338 patients included in the control group, 953 (71.23%) were categorized as non-infectious SIRS and 385 (28.77%) as normal healthy volunteers. The total proportion of patients with sepsis was 61.44%. There were 17 prospective studies and one retrospective. All were case–control design. Of these, 14 studies were defined as ‘gold standard’ by the criterions defined in the 1991 ACCP/SCCM consensus conference [42], two by the third international consensus definitions for sepsis and septic shock (sepsis-3) [43], one by Spanish Society of Infectious Diseases and Clinical Microbiology (SEIMC) [44], and another by American Burn Association (ABA) [45]. Among the types of specimen tested for presepsin detection, six studies used whole blood, ten studies used plasma, and two studies used serum. All studies used PATHFAST assay analysis system.

**Table 1:** Main characteristics of the included studies

References	Country	Mean age (years) total or case/control	Patient source	Sepsis/control (*n*)	Specimen tested	Inclusion criteria	Controls	Reference	Tests
Shozushima et al. [17]	Japan	62	CCU and ED	101/41	Whole blood	Presented to CCU and ED with ≥ 2 criteria for SIRS	SIRS (n = 41)	ACCP/SCCM 1991	PATHFAST
Endo et al. [29]	Japan	75/66	ICU and ED	115/70	Whole blood	Presented to ICU or ED with 1 criteria for SIRS	Non-infectious (n = 70)	Blood culture	PATHFAST
Liu et al. [33]	China	71/69	ED	680/279	Whole blood	Presented to ED with definition of sepsis by ACCP/SCCM	SIRS (n = 179) HC (n = 100)	ACCP/SCCM 2001	PATHFAST
Ulla et al. [38]	Italy	71/56	ED	106/83	Plasma	Presented to ED with ≥ 2 criteria for SIRS	SIRS (n = 83)	ACCP/SCCM 1991	PATHFAST
Vodnik et al. [39]	Serbia	56/55.15	ED	30/100	Plasma	Presented to ED with ≥ 2 criteria for SIRS and intra-abdominal infection	SIRS (n = 30) HC (n = 70)	ACCP/SCCM 1991	PATHFAST
Behnes et al. [25]	Germany	68/64	ICU	81/15	Serum	Presented to ICU with proven criteria of septic shock	SIRS (n = 15)	ACCP/SCCM 1991	PATHFAST
Brenner et al. [26]	Germany	70/44	ICU	60/60	Plasma	Presented to ICU with proven criteria of septic shock	HC (n = 30) Postoperative (n = 30)	ACCP/SCCM 2001	PATHFAST
Romualdo et al. [35]	Spain	71/67	ED	37/189	Plasma	Presented to ED with SIRS and suspected infection	Negative blood cultures in SIRS (n = 189)	SSIDCM	PATHFAST
Ishikura et al. [31]	Japan	67.2	ED	43/39	Whole blood	Presented to ED and CCM with ≥ 1 criteria for SIRS	Non-sepsis (n = 39)	ACCP/SCCM 1991	PATHFAST
Kweon et al. [32]	Korea	64.3/56.1	ED	73/45	Whole blood	Presented to ED with ≥ 2 criteria for SIRS	SIRS (n = 20) HC (n = 25)	ACCP/SCCM 1991	PATHFAST
Cakir Madenci et al. [27]	Turkey	38.5/44	ICU	26/11	Plasma	Presented to ICU with burn	Burn patients without sepsis (n = 11)	ABA	PATHFAST
Nakamura et al. [34]	Japan	70	ICU	37/75	Serum	ICU admission	Non-sepsis (n = 75)	ACCP/SCCM 1991	PATHFAST
Sargentini et al. [36]	Italy	55/53	CCU	60/44	Plasma	CCU admission	SIRS (n = 14) HC (n = 30)	ACCP/SCCM 1991	PATHFAST
Godnic et al. [30]	Slovenia	Not provided	ICU	40/7	Plasma	Presented to ICU with ≥ 2 criteria for SIRS	SIRS (n = 7)	Blood culture	PATHFAST
Takahashi et al. [37]	Japan	80	ED and ICU	359/97	Whole blood	Presented to ED with ≥ 1 criteria for SIRS	SIRS (n = 97)	ACCP/SCCM 1991	PATHFAST
Carpio et al. [28]	Peru	34/69	ICU	114/9	Plasma	Presented to ICU with SIRS and suspected infection	SIRS (n = 9)	ACCP/SCCM 1991	PATHFAST
Kada Klouche et al. [41]	France	58	ICU	100/44	Plasma	Presented to ICU with suspected infection	SIRS (n = 44)	Sepsis 3.0	PATHFAST
Romualdo et al. [40]	Spain	73/69	ED	70/130	Plasma	Presented to ED with suspected infection	Non-complicated infection (n = 130)	Sepsis 3.0	PATHFAST

*ABA* American Burn Association, *ACCP* American College of Chest Physicians, *ED* emergency department, *HC* healthy control, *ICU* intensive care unit, *postoperative control* major abdominal surgery without any evidence of infection, *SCCM* Society of Critical Care Medicine, *SIRS* systemic inflammatory response syndrome, *SSIDCM* Spanish Society of Infectious Diseases and Clinical Microbiology

**Table 2:** Diagnostic accuracy of the included studies

References	Thresholds (pg/mL)	AUC	Sensitivity	Specificity	TP	FP	FN	TN
Shozushima et al. [17]	415	0.85	0.80	0.81	81	8	20	33
Endo et al. [29]	600	0.91	0.88	0.81	101	13	14	57
Liu et al. [33]	317	0.82	0.71	0.86	481	40	199	239
Ulla et al. [38]	600	0.70	0.79	0.62	84	32	22	51
Vodnik et al. [39]	630	1.00	1	0.98	30	2	0	98
Behnes et al. [25]	700	0.84	0.90	0.60	73	6	8	9
Brenner et al. [26]	825	0.83	0.91	0.33	55	40	5	20
Romualdo et al. [35]	729	0.75	0.81	0.63	30	70	7	119
Ishikura et al. [31]	647	0.89	0.93	0.76	40	9	3	30
Kweon et al. [32]	430	0.94	0.88	0.82	64	8	9	37
Cakir Madenci et al. [27]	542	0.83	0.77	0.76	20	3	6	8
Nakamura et al. [34]	670	0.78	0.70	0.81	26	14	11	61
Sargentini et al. [36]	600	0.89	0.86	0.72	52	12	8	32
Godnic et al. [30]	413	0.71	0.85	0.63	34	3	6	4
Takahashi et al. [37]	685	0.89	0.80	0.83	285	16	74	81
Carpio et al. [28]	370	0.74	0.81	0.75	92	2	22	7
Kada Klouche et al. [41]	466	0.75	0.90	0.55	90	20	10	24
Romualdo et al. [40]	849	0.78	0.67	0.81	47	25	23	105

*TP* true positive, *FP* false positive, *FN* false negative, *TN* true negative, *AUC* area under the receiver operating characteristic curve

### Results of quality assessment

**Fig. 2:**
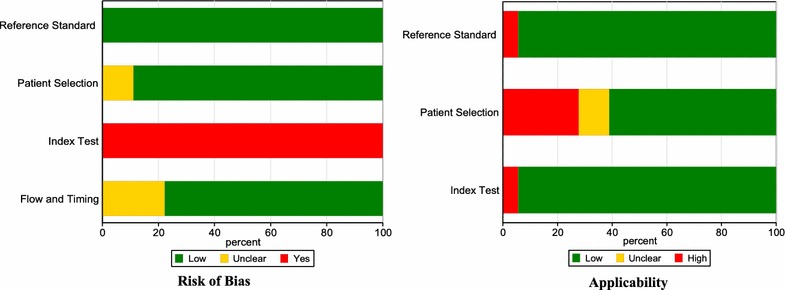
Graphical display of 18 studies results (QUADAS-2)

The methodological quality assessments with the QUADAS-2 tool for the 18 included studies are summarized in Table 3 and Fig. 2. All studies scored ‘low’ in the domain of bias in the reference standard, since the guidelines of the ACCP/SCCM consensus conference [42], Sepsis-3 [43], SEIMC [44], and ABA [45] were used to diagnose sepsis in these studies. For the domain of risk of bias in patient selection, the 16 studies providing clear definition of exclusion criteria were scored ‘low’ risk. Two studies that did not show enough information about how they excluded patients was scored ‘unclear’. Regarding the risk of bias in index tests, all studies not pre-specifying a threshold were scored ‘high risk’. For patient flow and timing domain, 14 studies scored ‘low’ since they clearly defined the appropriate interval between the index test and reference standard in their studies. In relation to applicability, all included studies scored well for the reference standard domain except one [35], since the target condition as defined by the reference standard (SEIMC) which was used for definition of bacteremia does not match the review question. In the patient selection criteria, 11 studies were in accordance with our inclusion criteria and scored ‘low’. In the quality assessment, the Cohen’s kappa statistic for the inter-rater agreement was 0.34. Subsequently, all disagreeing evaluations were resolved after consensus meetings.

**Table 3:**
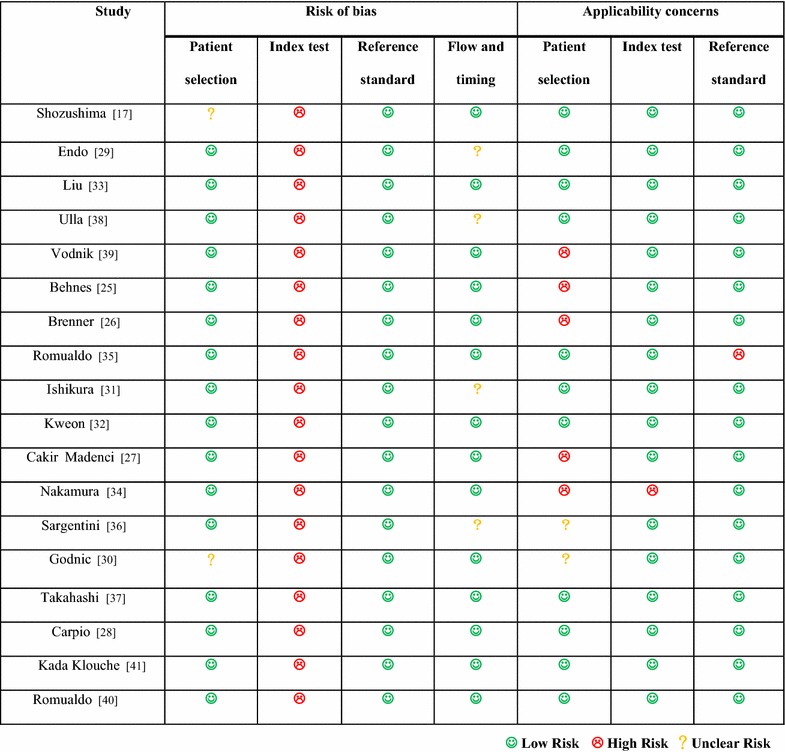
Quality assessment for 18 studies (QUADAS-2)

### Meta-analysis

**Fig. 3:**
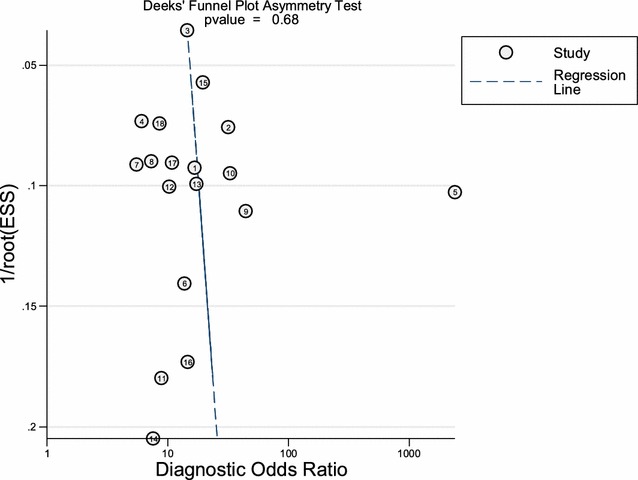
Deeks’ funnel plot asymmetry test for publication bias

**Fig. 4:**
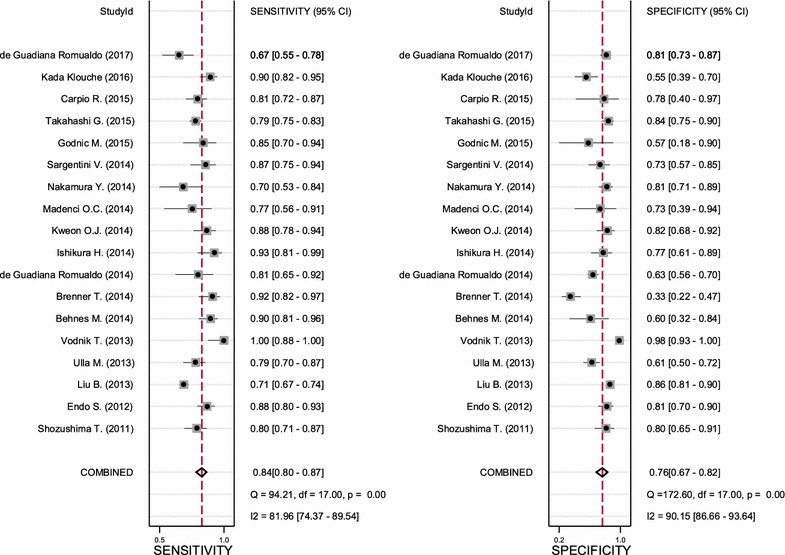
Forest plots of the sensitivity and specificity for presepsin across all included studies

**Fig. 5:**
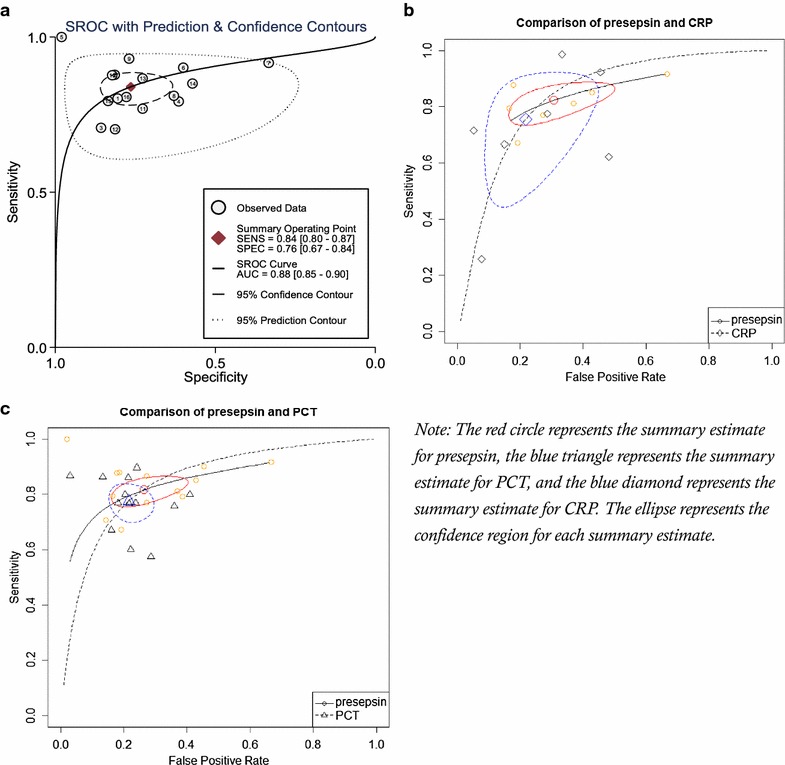
**a** Hierarchical summary receiver operating characteristic plot of presepsin across all included studies. **b** Comparisons of presepsin and C-reactive protein with summary receiver operating characteristic curves. **c** Comparisons of presepsin and procalcitonin with summary receiver operating characteristic curves. *Note* the *red circle* represents the summary estimate for presepsin, the *blue triangle* represents the summary estimate for procalcitonin, and the *blue diamond* represents the summary estimate for C-reactive protein. The *ellipse* represents the confidence region for each summary estimate

Deek’s effective sample size funnel plot and the regression test of asymmetry of the included studies indicated that there was no direct evidence for publication bias (*p* value = 0.68) (Fig. 3). The sensitivity of presepsin ranged from 0.67 to 1.0 among the 18 included studies, while the specificity of presepsin ranged from 0.33 to 0.98 (Fig. 4). The pooled sensitivity and specificity obtained by the HSROC method were 0.84 (95% CI 0.80–0.87) and 0.76 (95% CI 0.67–0.84), respectively (Figs. 4, 5a). We also constructed summary ROC for presepsin, and the result showed that the AUC was 0.88 (95% CI 0.85–0.90, Fig. 5a). The pooled DOR, PLR, and NLR of presepsin were 16 (95% CI 10–25), 3.4 (95% CI 2.5–4.6), and 0.22 (95% CI 0.17–0.27), respectively. The median cut-off for presepsin in the included studies was 600 pg/ml (IQR 439–664). More cautiously, after excluding one study [39] because its individual sensitivity and specificity were outside the confidence region in the summary operating point, we summarized the pooled statistics for remaining 17 studies [17, 25–38, 40, 41]. However, the subsequently pooled performance indices were not significantly different [sensitivity: 0.83 (95% CI 0.79–0.86), specificity: 0.72 (95% CI 0.65–0.79), AUC: 0.86, and pooled DOR: 13 (95% CI 10–17)]. Our analysis indicated that presepsin could offer a good degree of accuracy to diagnose sepsis.

### Investigation of heterogeneity

**Fig. 6:**
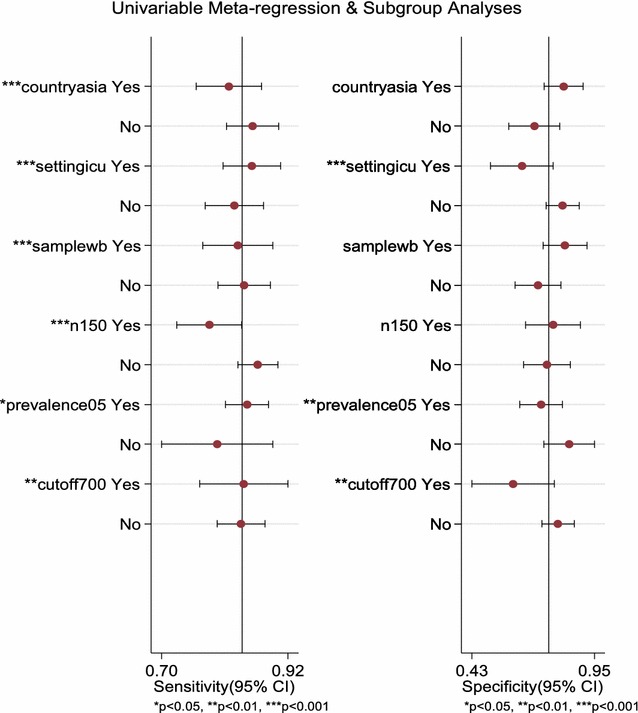
Plot of univariable meta-regression and subgroup analysis

From visual inspection of the SROC curve (Fig. 5a) and the estimation of the Spearman correlation coefficient (*ρ* = −0.26, *p* = 0.31), we concluded that heterogeneity was unlikely due to the diagnostic threshold effect. Significant heterogeneity in both sensitivities (Cochrane *Q* = 137.43, *p* < 0.001, *I* ^2^ = 87.63%) and specificities (Cochrane *Q* = 180.76, *p* < 0.001, *I* ^2^ = 90.60%) were found among all included studies for the non-threshold effect. The results of univariable meta-regression revealed that studies conducted in Asia, patients admitted in intensive care units, studies with whole blood specimens, sample sizes greater than 150, proportion of patients with sepsis greater than 0.5, and cut-off values greater than 700 accounted for the heterogeneity of sensitivity, whereas patient admitted in intensive care units, percentage of patients with sepsis great than 50%, and cut-off values great than 700 accounted for the heterogeneity of specificity (Table 4; Fig. 6).

**Table 4:** Summary of subgroup analysis of the included studies by the potentially influencing variables

Variable	Category	Number of studies	Number of patients	Pooled sensitivity	Pooled specificity
Asian country	Yes	8	2091	0.81 (0.76–0.86)	0.82 (0.78–0.85)
	No	10	1379	0.86 (0.80–0.90)	0.70 (0.54–0.83)
Patients admitted in ICU	Yes	8	783	0.86 (0.80–0.90)	0.64 (0.51–0.76)
	No	10	2687	0.83 (0.76–0.89)	0.82 (0.73–0.88)
Whole blood specimen	Yes	6	1942	0.83 (0.73–0.88)	0.82 (0.78–0.86)
	No	12	1528	0.84 (0.79–0.89)	0.71 (0.58–0.82)
Sample sizes great than 150	Yes	6	2215	0.78 (0.72–0.83)	0.77 (0.68–0.84)
	No	12	1255	0.87 (0.82–0.90)	0.75 (0.62–0.84)
Proportions of patients with sepsis great than 0.5	Yes	14	2802	0.84 (0.80–0.88)	0.72 (0.63–0.79)
	No	4	668	0.85 (0.55–0.96)	0.86 (0.63–0.95)
Cut-off value great than 700	Yes	4	642	0.85 (0.70–0.93)	0.59 (0.37–0.78)
	No	14	2828	0.84 (0.79–0.87)	0.80 (0.72–0.85)

### Results of subgroup analyses

In subgroup analyses, we found lower pooled sensitivity and higher pooled specificity in the Asian countries (0.81 vs. 0.86, *p* < 0.05; 0.82 vs. 0.70, *p* < 0.05, respectively, Table 4). Furthermore, the higher pooled specificity in the non-ICU settings (0.82 vs. 0.64), whole blood specimens (0.82 vs. 0.71), lower than 50% of patients with sepsis (0.86 vs. 0.72), cut-off values great than 700 (0.80 vs. 0.59, all *p* < 0.05, Table 4) were found.

Seven of the included studies [31–33, 35, 38–40], comprising 1904 patients, were performed in ED, and eight [25–28, 30, 34, 36, 41], comprising 783 patients, were in ICU (Table 1). We performed a subgroup analysis restricted to these two groups because of the possibility that different levels of severity existed between them. The pooled specificity was higher in ED versus ICU (0.82, 95% CI 0.69–0.91 vs. 0.64, 95% CI 0.51–0.76). The AUC indicated the possible better accuracy of presepsin in ED than in ICU (0.91 vs. 0.85).

### Different compositions of control group

Among the included studies, five studies [27, 29, 31, 34, 39] recorded the diagnosis accuracy between healthy control and sepsis, eight studies [17, 28, 30, 35, 37, 38, 40, 41] recorded it between non-infectious SIRS and sepsis, and five studies [25, 26, 32, 33, 36] recorded it between mixed controls (SIRS and normal) and sepsis. We found the higher pooled sensitivity, specificity, AUC, and DOR for the first group (0.88, 0.82, 0.88, 26, respectively, Table 5). The cut-off values for presepsin used in these three groups ranged from 542–670, 370–849, and 317–825 pg/ml, respectively, and the median cut-off was 618 pg/ml (IQR 600–647), 533 pg/ml (IQR 415–696), and 574 pg/ml (IQR 430–700), respectively.

**Table 5:** Summaries of pooled sensitivity and specificity of presepsin to diagnose sepsis for different compositions of control group

Case	Control	Number of studies	Number of patients	Pooled sensitivity	Pooled specificity	AUC	Pooled DOR	Median cut-off
Sepsis	Healthy	5	546	0.88 (0.70–0.96)	0.82 (0.75–0.88)	0.88	26 (9–75)	618
Sepsis	Mixed	5	1397	0.86 (0.76–0.92)	0.68 (0.47–0.83)	0.86	15 (9–23)	574
Sepsis	Non-infectious SIRS	8	1527	0.81 (0.76–0.85)	0.71 (0.63–0.78)	0.84	10 (8–15)	533

### Performance comparison with PCT and CRP

The summary diagnostic accuracy of the included studies for biomarkers, presepsin, PCT, and CRP is summarized in Table 6. From Table 7 and Fig. 5b, c, we found no significant difference between presepsin and PCT in 13 studies comprising 2915 patients. Furthermore, the pooled sensitivity of presepsin was found to be higher than PCT in 5 studies conducted in ICU comprising 452 patients (0.88, 95% CI 0.82–0.92 vs. 0.75, 95% CI 0.68–0.81), while the pooled specificity of presepsin was lower than PCT (0.58, 95% CI 0.42–0.73 vs. 0.75, 95% CI 0.65–0.83). Additionally, the analysis results revealed that the AUC of presepsin was similar with CRP (0.85 vs. 0.85) in seven studies comprising 1204 patients.

**Table 6:** Summary diagnostic accuracy of the included studies for biomarkers, presepsin, procalcitonin, and C-reactive protein

References	Sepsis/control (*n*)	Presepsin			Procalcitonin			C-reactive protein		
		Sensitivity	Specificity	AUC	Sensitivity	Specificity	AUC	Sensitivity	Specificity	AUC
Shozushima et al. [17]	101/41	0.80	0.81	0.88	–	–	0.65	–	–	0.82
Endo et al. [29]	115/70	0.88	0.81	0.91	0.86	0.79	0.91	–	–	–
Liu et al. [33]	680/279	0.71	0.86	0.82	0.60	0.78	0.72	–	–	–
Ulla et al. [38]	106/83	0.79	0.62	0.70	0.89	0.76	0.88	–	–	–
Vodnik et al. [39]	30/100	1	0.98	0.99	0.87	0.97	0.96	–	–	0.96
Behnes et al. [25]	81/15	0.90	0.60	0.84	–	–	0.86	–	–	–
Brenner et al. [26]	60/60	0.91	0.67	0.83	0.77	0.78	0.84	0.67	0.85	0.79
Romualdo et al. [35]	37/189	0.81	0.63	0.75	0.76	0.64	0.79	0.62	0.52	0.60
Ishikura et al. [31]	43/39	0.93	0.76	0.89	–	–	–	–	–	–
Kweon et al. [32]	73/45	0.88	0.82	0.94	0.86	0.87	0.92	0.99	0.67	0.85
Cakir Madenci et al. [27]	26/11	0.77	0.76	0.83	0.76	0.79	0.85	0.92	0.58	0.82
Nakamura et al. [34]	37/75	0.70	0.81	0.78	–	–	–	–	–	–
Sargentini et al. [36]	60/44	0.86	0.72	0.89	0.80	0.80	0.91	–	–	–
Godnic et al. [30]	40/7	0.85	0.63	0.71	0.58	0.67	0.63	0.77	0.75	0.73
Takahashi et al. [37]	359/97	0.80	0.83	0.89	0.77	0.76	0.85	0.72	0.95	0.82
Carpio et al. [28]	114/9	0.81	0.75	0.83	–	–	–	–	–	–
Kada Klouche et al. [41]	100/44	0.90	0.55	0.75	0.80	0.59	0.80	–	–	–
Romualdo et al. [40]	70/130	0.67	0.81	0.78	0.67	0.84	0.82	0.26	0.92	0.59

AUC, area under the receiver operating characteristic curve; –, not report

**Table 7:** Summaries of performance statistics of presepsin, procalcitonin, and C-reactive protein for diagnosing sepsis

Category	Biomarkers	Number of studies	Number of patients	Pooled sensitivity	Pooled specificity	AUC	Pooled DOR
All	Presepsin	13	2915	0.84 (0.79–0.88)	0.75 (0.64–0.84)	0.87 (0.84–0.90)	16 (8–30)
	Procalcitonin	13	2915	0.78 (0.72–0.83)	0.79 (0.73–0.85)	0.86 (0.82–0.88)	14 (8–23)
All	Presepsin	7	1204	0.83 (0.76–0.88)	0.70 (0.55–0.81)	0.85 (0.81–0.88)	11 (7–18)
	C-reactive protein	7	1204	0.77 (0.53–0.91)	0.79 (0.62–0.89)	0.85 (0.82–0.88)	13 (5–36)
ED	Presepsin	6	1822	0.83 (0.68–0.92)	0.83 (0.67–0.92)	0.90 (0.87–0.92)	24 (5–113)
	Procalcitonin	6	1822	0.79 (0.68–0.87)	0.83 (0.72–0.91)	0.88 (0.85–0.90)	19 (7–52)
ICU	Presepsin	5	452	0.88 (0.82–0.92)	0.58 (0.42–0.73)	0.87 (0.84–0.90)	10 (5–18)
	Procalcitonin	5	452	0.75 (0.68–0.81)	0.75 (0.65–0.83)	0.82 (0.78–0.85)	9 (6–15)
Sepsis-1/2	Presepsin	7	2076	0.86 (0.76–0.91)	0.79 (0.60–0.91)	0.90 (0.87–0.92)	23 (7–77)
	Procalcitonin	7	2076	0.80 (0.72–0.87)	0.83 (0.75–0.89)	0.89 (0.86–0.91)	20 (9–45)
Sepsis-1/2	Presepsin	3	694	0.86 (0.75–0.93)	0.69 (0.33–0.90)	0.87 (0.83–0.91)	16 (6–38)
	C-reactive protein	3	694	0.86 (0.44–0.98)	0.85 (0.63–0.95)	0.91 (0.86–0.93)	34 (9–127)

Among these 13 studies that have a direct comparison between presepsin and PCT, seven studies defined the ‘gold standard’ by the criterions defined in the 1991 ACCP/SCCM consensus conference. The results revealed that the performance of presepsin was similar with PCT in these seven studies (Table 7). Similarly, among the seven studies that compared presepsin with CRP, there were three studies that utilized the criterions defined in the 1991 ACCP/SCCM consensus conference as ‘gold standard’, and the results revealed that CRP has higher pooled specificity (0.85 vs. 0.69) and AUC (0.91 vs. 0.87) than presepsin. However, the study numbers utilizing other criteria (sepsis 3.0, ABA, SEIMC) were too small to perform the subgroup meta-analysis.

### Optimal cut-off

**Fig. 7:**
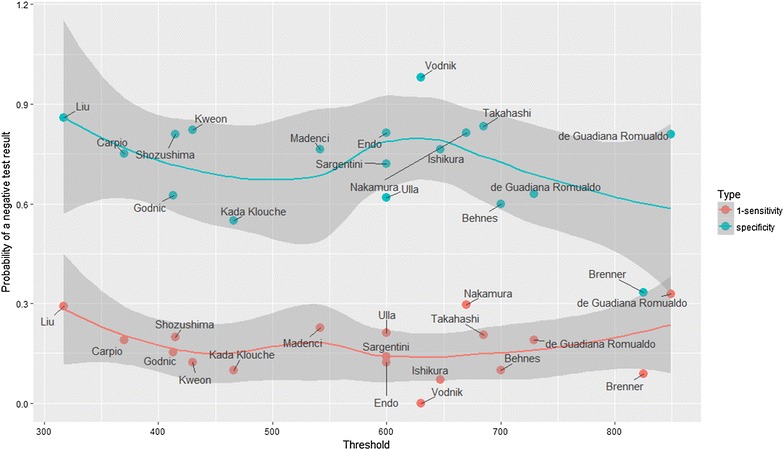
Plot for solve the ‘optimal cut-off’

From Fig. 7, the difference between these two fitted lines, known as Youden index, varied by different cut-offs. The cut-off that maximized the Youden index was 600–650 pg/ml. Considering the varied cut-off values between studies, we calculated the pooled sensitivity and specificity for four studies [25, 26, 35, 40] with cut-off values great than 700 pg/ml and for the other 14 studies [17, 27–34, 36–39, 41] that cut-off value smaller than 700 pg/ml. We found the lower pooled specificity in the cut-off values greater than 700 pg/ml studies (0.59 vs. 0.80, *p* < 0.05, respectively, Table 4).

## Discussion

As sepsis is the leading cause of death among critically ill patients, early diagnosis of sepsis is often essential for the following treatment to improve outcomes. Since clinical signs of sepsis often overlap with systemic inflammatory response syndrome (SIRS) [12], an effective biomarker is needed to distinguish sepsis from non-infectious SIRS. Presepsin, which is part of CD14, can be detected in the blood and its level elevates closely related to the immune response to LPS [16]. A previous study also found the level of presepsin measured from patients with infection could be well distinguished from those measured from patients without infection, which has indicated that presepsin might be a promising biomarker in diagnosing sepsis from the physiological aspect [29].

To our knowledge, there have been five meta-analyses published recently evaluating the diagnostic value of presepsin [46–50]. However, we found that some of them were designed sub-optimally regarding study eligibility. In addition, in their subgroup analyses revealed strong heterogeneity due to small samples sizes and lower percentages of patients with sepsis between studies. Moreover, most of them mentioned the requirement of comparison with other biomarkers. Our meta-analysis not only included more recently studies but also compared presepsin with other biomarkers (PCT and CRP). Additional subgroup analyses were also conducted due to high heterogeneity between studies. Besides, potential influences caused by different compositions of control groups and different timings when biomarkers were measured were also assessed.

After reviewing 18 studies, a total of up to 3470 patients were included. Our results suggested good overall diagnostic accuracy for presepsin according to the pooled sensitivity and specificity and AUC value. As it is often not easy to give a clinical explanation for a test result directly from the AUC value, which indicates an overall accuracy for diagnosis, we provided a more understandable clinical explanation of the results of the pooled positive likelihood ratio (PLR: 3.4) and the negative likelihood ratio (NLR: 0.22). The clinical explanation for PLR and NLR suggested that it could be nearly four times more likely for a patient to have sepsis with a positive presepsin result and only one-fourth for a patient with a negative presepsin result. However, further studies for the diagnostic performance of presepsin were still needed due to a few possible issues found for presepsin according to the results of our subgroup analyses.

The different definitions of control groups could be one of the possible sources of heterogeneity among those included studies. Instead of distinguishing sepsis from normal healthy controls, the methods to distinguish non-infectious SIRS from sepsis were truly necessary in the real world clinical situation. However, normal healthy controls might be included in the clinical trials for good diagnostic outcomes. From our result (see Table 5), we found lower diagnostic accuracy when leaving out the normal healthy controls from control groups (AUC value 0.84). Consequently, the reliability of the pooled result could be called into question. Therefore, we recommended that future studies avoid the case–control design as QUADAS-2 suggested [21].

Another possible source of heterogeneity came from the use of different specimen types. Our subgroup analysis result suggested that the use of whole blood had higher specificity than plasma, with statistical significance. Nevertheless, a simple adjustment might be sufficient enough to eliminate the bias. A previous study revealed that, when measured using PATHFAST system, cardiac troponin I (cTnI), myoglobin, MB isoenzyme of creatine kinase (CK–MB), and N-terminal probrain natriuretic peptide (NT-proBNP) were shown to have a highly correlated linear relationship (correlation coefficients >0.9, calculated by Passing–Bablok regression analysis) between two types of samples [51]. However, whether there is also a linear relationship existing between two types of sampling methods for presepsin is still not confirmed. Therefore, we suggested future studies to examine the relationship for this possible bias.

Interestingly, we found lower pooled specificity among patients who were admitted to the ICUs. We suggest that higher proportion of critically ill patients in the ICU might be a possible source. However, this finding could be difficult to explain clinically, since it could come from numerous other possible sources. More information is needed to explore this result in more detail.

PCT is a biomarker currently used for diagnosis of sepsis [9, 10], and its diagnostic accuracy has been widely studied. From a meta-analysis of PCT in 2007 [11], PCT seemed to become widely studied since 1999 [52, 53], while the earliest study we found for presepsin was in 2011 [17]. This might indicate that presepsin was still not a well-studied biomarker compared to PCT. In addition, a previous study revealed that the presepsin level typically elevates earlier than PCT [14]. We have tried to replicate the result by performing a subgroup analysis to compare the diagnostic accuracy of presepsin with PCT in the ED, which approximately represented the earlier stage of sepsis. However, we did not find any significant difference between presepsin and PCT measured in either EDs (AUC 0.90 and 0.88) or ICUs (AUC 0.87 and 0.82). Besides, from our subgroup analysis result for these studies, we found similar diagnostic accuracy between presepsin (AUC 0.87) and PCT (AUC 0.86), which indicates that presepsin is a promising biomarker for study. Our current results suggested no obvious better performance of presepsin than PCT in the diagnosis of sepsis. Further studies focusing on the diagnostic accuracy difference between presepsin and PCT might still be needed.

Although CRP is another commonly used biomarker in the clinical context, previous studies revealed that its diagnostic accuracy for sepsis is significantly lower than PCT [11]. But, after comparing presepsin with CRP, we observed no significant difference between CRP (AUC 0.85) and presepsin (AUC 0.85). Furthermore, the confidence region overlapped from the sensitivity versus FPR curve is summarized in Fig. 5b, c. Our results supported that presepsin showed similar performance with CRP in sepsis diagnosis.

The strengths of this systematic review and meta-analysis include (1) the performance of presepsin was formally compared with PCT and CRP; (2) detailed subgroup analyses were utilized to solve the heterogeneity between the included studies; (3) different compositions of control groups (healthy control, non-infectious SIRS, and mixed) were compared in the subgroup analysis; (4) the timing of biomarker measurements and different specimen types were taken into account in the analysis; and (5) the optimal cut-off was attempted to be determined in this study.

There were some limitations for our study. First, most studies we included diagnosed sepsis using reference standard from ACCP/SCCM 1991 (10 studies) and 2001 (2 studies) (Sepsis 1.0 or 2.0) instead of the newly defined Sepsis 3.0 [43]. Clinical trials are still needed to re-evaluate the performance and the optimal cut-off of presepsin accordingly. Second, although we have included 18 studies in our study, the statistical power might still be not enough to confirm the diagnostic value of presepsin. Finally, as we only include studies written in English, there may be some language bias in our study.

## Conclusions

Based on the results of our meta-analysis, presepsin is a promising marker for diagnosis of sepsis as PCT or CRP, but these results should be interpreted carefully and cautiously, since only a limited number of studies included and high heterogeneity between them. Additionally, it cannot be recommended as a single test for sepsis diagnosis, but may be useful in combination with some sensitive biological markers. In addition, continuing re-evaluation during the course of sepsis is advisable.

## Abbreviations

ABA: American Burn Association
AUC: area under the receiver operating characteristic curve
CRP: C-reactive protein
CI: confidence interval
DOR: diagnostic odds ratio
ED: emergency department
HSROC: hierarchical summary receiver operating characteristic
ICU: intensive care unit
LBP: lipopolysaccharide-binding protein
LPS: lipopolysaccharide
LR: likelihood ratio
NLR: negative likelihood ratio
PCT: procalcitonin
PLR: positive likelihood ratio
QUADAS: Quality Assessment of Diagnostic Accuracy Studies
SEIMC: Spanish Society of Infectious Diseases and Clinical Microbiology
SIRS: systemic inflammatory response syndrome
SROC: summary receiver operating characteristic
TREM-1: myeloid cells expressing triggering receptor-1
